# Selenium‐Based Molecules to Treat Alzheimer’s Disease

**DOI:** 10.1002/alz70859_103404

**Published:** 2025-12-25

**Authors:** Praveen Nekkar Rao, Ahmed Abdallah Hefny, Rahul C Karuturi, Arash Shakeri

**Affiliations:** ^1^ University of Waterloo, Waterloo, ON Canada

## Abstract

**Background:**

The neurodegenerative disorder Alzheimer’s disease (AD), is the major cause of dementia. As per the World Health Organization, >55 million people are affected with dementia worldwide. The health and economic burden of AD will only increase in the coming years due to the lack of effective and curative therapies. In this regard, the recent market launch of monoclonal antibodies lecanemab and donanemab to treat AD is an exciting development for patients and caregivers. This has renewed the interest in targeting the amyloid‐beta (Aβ) as a viable strategy to treat AD. In this regard, designing small molecule therapies to prevent the aggregation and neurotoxicity of Aβ has the potential to discover novel anti‐AD therapies.

**Method:**

The selenium‐based N‐benzylphenoselenazines were designed by investigating their interactions with Aβ40 assemblies and to incorporate suitable substituents. The N‐benzylphenoselenazines library synthesis was carried out by optimizing the synthetic chemistry protocols. The synthesized compounds were characterized by analytical methods. The in vitro anti‐Aβ40 activity was evaluated using thioflavin‐T based fluorescence aggregation kinetics and electron microscopy studies. The antioxidant activity was also evaluated in vitro. The cytotoxicity was evaluated in mouse hippocampal HT22 neuronal cells. The blood‐brain barrier permeability of novel N‐benzylphenoselenazine derivatives was also evaluated.

**Result:**

The compound library synthesis was carried out by coupling diphenylamines with selenium to obtain the phenoselenazine scaffold which was treated with benzyl halides to obtain the N‐benzylphenoselenazines (yield = 23–89%). The analysis of the compound library by ^1^H, ^13^C NMR, LCMS and HRMS confirmed their chemical structures and sample purity. In vitro studies demonstrated their Aβ40 aggregation inhibition activity ranging from 26–85% which was further confirmed by electron microscopy studies. Compounds from this series also demonstrated antioxidant activity ranging from 23–80.5%. Furthermore, these compounds were not toxic to hippocampal HT22 neuronal cells.

**Conclusion:**

This study led to the identification of novel selenium‐based small molecules (N‐benzylphenoselenazines) that are able to reduce Aβ40 aggregation and demonstrate antioxidant activity suggesting their potential application as disease‐modifying agents to treat AD.

